# Effect of berberine on cognitive function and β-amyloid precursor protein in Alzheimer’s disease models: a systematic review and meta-analysis

**DOI:** 10.3389/fphar.2023.1301102

**Published:** 2024-01-16

**Authors:** Jia-Yang Liu, Yu Dai, Yao-Xi He, Lin Lin

**Affiliations:** ^1^ School of Elderly Health, Chengdu Medical College, Chengdu, Sichuan, China; ^2^ School of Nursing, Chengdu Medical College, Chengdu, Sichuan, China; ^3^ Chengdu Eighth People’s Hospital (Geriatric Hospital of Chengdu Medical College), Chengdu, Sichuan, China; ^4^ Sichuan Collaborative Innovation Center for Elderly Care and Health, Chengdu, Sichuan, China

**Keywords:** berberine, cognitive function, β-amyloid precursor protein, Alzheimer’s disease, animal models, meta-analysis

## Abstract

**Introduction:** Berberine is an isoquinoline alkaloid extracted from Berberis vulgaris, which possesses a variety of pharmacological activities. Alzheimer’s disease (AD) is a complex disease with multiple pathologic factors, with cognitive decline being the main manifestation of AD. The neuroprotective effects of berberine in animal models of Alzheimer’s disease (AD) have been widely reported, exhibiting protective effects against risk factors associated with AD. In this study, we summarize and evaluate the effects of berberine on cognitive function and β-amyloid precursor protein in animal models of AD.

**Material and methods:** Eligible studies were retrieved from PubMed, MEDLINE, EMBASE, Web of Science, and Cochrane Library databases up to 1 June 2023. Risk of bias was assessed by the Systematic Review Center for Laboratory Animal Experiments (SYRCLE). Statistical analyses were performed using STATA 14.0 and Review Manger 5.4 software to calculate weighted standardized mean difference (SMD) and 95% confidence intervals (CI), Morris water maze (MWM) test and β-amyloid precursor protein as outcome measures. Heterogeneity was tested using the I^2^ test. Sensitivity analysis and publication bias were also assessed.

**Results:** 19 studies involving 360 animals met the inclusion criteria, and the results of the meta-analysis showed that berberine decreased escape latency (SMD = −2.19, 95% CI: (−2.50, −1.88), *p* < 0.00001), increased the number of platform crossings (SMD = 4.27, 95% CI (3.38, 5.17), *p* < 0.00001), time in the target quadrant (SMD = 5.92, 95% CI (4.43, 7.41), *p* < 0.00001) and APP expression (SMD = 0.73, 95% CI: (0.25, 1.21), *p* = 0.003).

**Conclusion:** Berberine can regulate APP expression and improve cognitive function in animal models of AD, and the mechanism may be related to the involvement of berberine in APP processing and influence the expression of its related factors.

**Systematic review registration:** PROSPERO, CRD42023437445

## 1 Introduction

Alzheimer’s disease (AD) is a neurodegenerative condition characterized by memory loss and cognitive decline. Currently, over 55 million individuals globally experience dementia, and this number is projected to surpass 78 million by 2030 due to population aging, with AD contributing to 60%–70% of dementia cases (Leng and Edison, 2021; Leng and Edison, 2021). It stands as the primary cause of disability among individuals aged 65 and above worldwide (Jia et al., 2020). It is swiftly emerging as one of this century’s most burdensome, costly, and lethal diseases (Scheltens et al., 2021). As the population aged 65 and above grows, so does the prevalence of AD among older adults. In summary, AD poses a significant global health threat (Dauphinot et al., 2022).

There are many typical pathological features of AD including accumulation of tau neurogenic fiber tangles (Busche and Hyman, 2020), synaptic degeneration (Griffiths and Grant, 2023), mitochondrial dysfunction (Morton et al., 2021) neuroinflammation and oxidative stress (Tonnies and Trushina, 2017) among others. One of the characteristic hallmarks of AD is the deposition of senile plaques containing β-amyloid (Aβ). Aβ is produced from the amyloid precursor protein (APP) by sequential proteolytic cleavage by β-secretase and γ-secretase. The aggregation of Aβ into amyloid plaques is considered a pivotal pathogenic event in AD. APP is a transmembrane precursor protein widely expressed in the central nervous system, peripheral tissues of the liver and pancreas, adipose tissue, and myotubes. APP is first cleaved by β-site cleaving enzyme 1 (BACE 1) *in vivo*, releasing soluble APPβ (sAPPβ) peptide (Kim and Tsai, 2009). APP peptides produced by non-amyloidogenic pathways (e.g., sAPPα) are metabolically beneficial in the central nervous system and peripheral tissues. However, amyloidogenesis triggered by APP cleavage not only exacerbates AD progression but also adversely affects metabolic conditions. Therefore, pharmacological modulation to increase non-amyloid pathway peptide production and reduce amyloidogenic pathway peptides, specifically targeting BACE1-mediated APP cleavage without impacting other substrates, might prove effective in combating AD (Guo et al., 2021b). Understanding the role of APP in AD pathogenesis concerning Aβ production and abnormal aggregation is crucial. However, current therapeutic strategies against β-amyloid have almost always ended in failure in clinical trials. The first new AD drug, aducanumab (Aduhelm), approved by the U.S. FDA using the accelerated approval pathway since 2003, has also generated much controversy (Liu and Howard, 2021), so it is necessary to search for better drugs for AD.

Donepezil, carboplatin, galantamine and memantine are routinely used in clinical practice for the treatment of AD. However, these medications are single-target drugs focusing on specific mechanisms, exhibiting temporary and modest symptomatic improvements but hardly preventing or reversing AD progression (Silva et al., 2014). Traditional Chinese medicine (TCM) is characterized by multi-targets, multi-systems, multi-links and multi-pathways in the treatment of dementia, which shows the unique advantages of TCM (Chen et al., 2020a). Berberine, a natural alkaloid, has become a hot research topic for its role in central nervous system diseases (Cheng et al., 2022). Berberine inhibits neuroinflammation, oxidation, and endoplasmic reticulum stress production, exhibiting neuroprotective, antioxidant, and anti-inflammatory properties, thereby reducing neuronal damage and apoptosis (Zhang et al., 2020). Multiple studies emphasize berberine’s efficacy in enhancing conditions related to cognitive impairment (Fang et al., 2020; Zhang et al., 2021a; Yao et al., 2023). Research indicates that berberine (BBR) curbs Aβ-induced microglia activity by modulating suppressor of cytokine signaling 1 (SOCS 1) (Guo et al., 2021a). It has also been shown that endoplasmic reticulum stress is central to signaling by the mechanism of phosphorylation of tau protein by hyperactivation of glycogen synthase kinase 3β (GSK 3 *β*) and phosphorylation of eukaryotic translation initiation factor-2α (eIF2α) by activation of PRKR-like endoplasmic reticulum kinase (PERK). Berberine ameliorates endoplasmic reticulum stress and thus cognitive deficits in APP/PS1 mice (Wu et al., 2021). While various experiments have assessed berberine’s anti-AD properties, there has not been a systematic review of its effects on cognitive function and β-amyloid precursor protein. Therefore, it is necessary to investigate the effects of berberine on cognitive function and β-amyloid precursor protein to clarify the therapeutic potential of berberine in AD.

Based on the limitations of current studies and systematic evaluations, the purpose of this study was to systematically review the current literature in evaluating the effects of berberine on the MWM test and in β-amyloid precursor proteins in AD models. Behavioral indicators were analyzed to determine whether berberine improves cognitive function in animal models, to further evaluate the efficacy of berberine on β-amyloid expression in AD models and the potential mechanisms, and to explore the different effects of different intervention times, modes of intervention, and dosages and routes of administration in mice with AD models.

## 2 Materials and methods

### 2.1 Search strategy

Qualified studies published through June 2023 were searched in PubMed, Embase, Web of Science, and the Cochrane Library. The strategy was developed based on a combination of medical subject headings (MeSH) and free-text terms, and the database search strategy was based on PubMed as an example, with the search formula shown below: (Alzheimer Disease [MeSH Terms]) OR (Dementia [MeSH Terms]) OR (Cognition [MeSH Terms]) OR (Alzheimer) OR (AD) OR (cognitive) AND “Berberine” (MeSH Terms) OR “Berberine” [Title/Abstract] OR “Umbellatine” [Title/Abstract]. The detailed search strategy was provided in the Supplementary Material.

### 2.2 Inclusion and exclusion criteria

#### 2.2.1 Inclusion criteria

Two investigators (JY-L and YX-H) jointly developed the inclusion and exclusion criteria for this review. Studies were considered if all of the following criteria were met: 1) an animal model with Alzheimer’s disease, induced by genetic variants (transgenics) or drugs; 2) berberine dosing was the only intervention, with no restriction on the duration of the intervention, dosage of the drug, mode of dosing, or type of mice; 3) availability of the Morris Water Maze (MWM) test or the β-amyloid precursor protein (APP) indicator; 4) randomized controlled trials; 5) published in English.

#### 2.2.2 Exclusion criteria

1) Therapies other than interventions using berberine; 2) studies of cytokines; 3) duplicate literature or literature without a control group; 4) lack of full text, literature review studies, course-completion theses, dissertations, theses, and abstracts for yearbooks; 5) literature that did not meet the inclusion criteria after manual screening was also excluded.

### 2.3 Data extraction

Two researchers (J-YL and Y-XH) independently extracted the following data and resolved differences by consulting with a third reviewer: 1) year of publication and name of the first author, 2) animal information, including species, sex, age, weight, and sample size of each group, etc. 3) intervention regimen, including duration of the intervention, dosage of medication, and mode of medication administration, etc. 4) assessment of the outcome, including the escape latency, the number of platform crossings, time in the target quadrant selected in the MWM test, and β-amyloid precursor protein, with the fourth day or more extracted as the final outcome if the outcome was presented at different time points. If the data were presented only graphically, the values in the graphs were estimated using the GetData Graph Digitizer 2.26.

### 2.4 Quality evaluation and risk of bias assessment

Two evaluators independently assessed the quality of the study using the 10-item scale introduced by the SYRCLE Risk of Bias (RoB) Tool for Animal Research and resolved differences by consulting a third reviewer. The evaluation criteria of the tool are: random allocation sequence; similar baseline characteristics; allocation concealment; randomized housing; blinded interventions; random selection for outcome assessment; blinded assessment of outcomes; incomplete outcome data; selective outcome reporting; and bias from other sources (Hooijmans et al., 2014). A “Y” response indicates a low risk of bias, an “N” response indicates a high risk of bias, and an “NC” response indicates an uncertain level of bias due to insufficient data. One point was awarded for each “Y” response.

### 2.5 Statistical analysis

Data were analyzed using RevMan 5.4 and Stata 14.0 software. A random effects model was used to calculate mean ± SD, 95% confidence intervals (CI) and standardized mean difference (SMD) to account for potential heterogeneity. The heterogeneity of the included data was determined by calculating the I^2^ value; if I^2^ ≤ 50%, *p* ≥ 0.1, it was considered that there was no significant heterogeneity among the studies, and the fixed-effects model was used for the analysis; if I^2^ > 50%, *p* < 0.1, it was considered that there was significant heterogeneity among the studies, and the random-effects model was used for the analysis; when the heterogeneity was higher, regression analysis, subgroup analysis, and sensitivity analysis were used to explore the source of heterogeneity, and funnel plot and Egger’s test were used to evaluate publication bias.

## 3 Results

**FIGURE 1:**
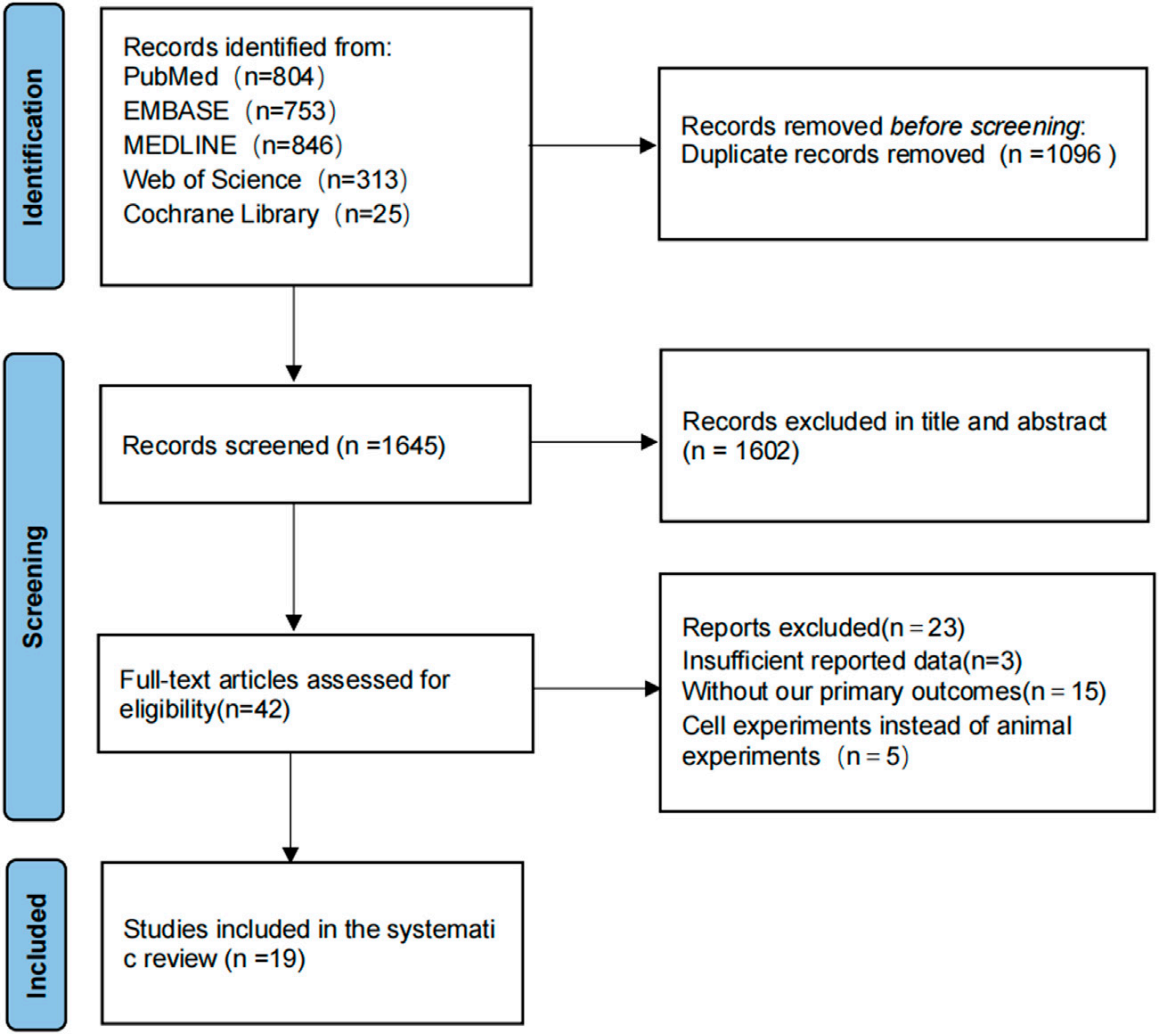
The flow diagram of the study selection process.

In accordance with the developed literature search, a total of 2,741 articles were found in the initial search for the item study strategy, and 1,096 duplicates were excluded through Notexpress literature management software, leaving 1,645 articles to be screened. A total of 1,602 articles were excluded by reading the title and abstract, 23 articles were excluded by reading the full text, and 23 studies were excluded due to the following reasons: incomplete study data; absence of the primary endpoints of our study; cellular experiments rather than animal experiments, and 19 articles were finally included in the final literature (Zhu and Qian, 2006; Bhutada et al., 2011; Durairajan et al., 2012; Mangrulkar et al., 2013; Patil et al., 2015; de Oliveira et al., 2016; Kumar et al., 2016; Chen et al., 2017; He et al., 2017; Huang et al., 2017; Cai et al., 2018; Hussien et al., 2018; Chen et al., 2020b; Lin et al., 2020; Wang et al., 2021b; Liang et al., 2021; Wu et al., 2021; Ye et al., 2021; Yang and Wang, 2022). The flow diagram of the selection process is shown in (Figure 1).

### 3.1 Characterization of the study

A total of 19 papers were included with a total of 360 mice, including 175 animals in the control group and 175 in the exercise group. AD models included both transgenic and non-transgenic models. Five studies used the APP/PS1 mouse model (n = 5). Four studies used the 3 × Tg AD mouse model (n = 4), five studies used Wistar rats (n = 5), two studies used Sprague-Dawley rats (n = 2), one study used TgCRND8 mice, one study used Swiss Albino mice and one study used B6C3-Tg mice. Thirteen studies used only male animals, one study used only female animals, two studies used animals of both sexes, and three did not describe the sex of the animals used in the experiment. The duration of the intervention ranged from half a month to 4 months. A total of 14 out of 19 studies mentioned the age of the animals ranging from 1 month to 8 months of age. Seventeen studies used dosing doses of 50 mg–100 mg/kg, one study used a dose of 40 mg/kg, and one study used a dose of 187.5 mg/kg. Outcome indicators: 17 studies mentioned escape latency, 14 studies mentioned duration in the platform quadrant, 13 studies mentioned the number of platform crossings, 8 studies mentioned APP. All these data can be found in Table 1.

**TABLE 1:** Basic information of included studies.

Study	Animal models (modeling age, weight, sex, n = treatment/model group)	Berberine intervention (dosage, route and time)	Outcome measures
Bhutada et al. (2011)	Wistar rats model of streptozotocin-induced diabetes (200–225 g, male, n = 6/6)	25/50/100 mg/kg, orally, 30 days	①②③
Cai et al. (2018)	APP/PS1 transgenic mice (The 120-day, male, n = 10/10)	50/100 mg/kg, intragastric administration, 14 days	①②③④
Chen et al. (2017)	Wistar rats model of streptozotocin-induced diabetes (4–5 weeks, 200 g, male, n = 3/3)	187.5 mg/kg/d, intragastric administration, 28 days	④
Chen et al. (2020b)	The 3 × Tg AD mice (male, n = 12/12)	100 mg/kg/day, orally, 4 months	①②③
de Oliveira et al. (2016)	ICV-STZ induced sporadic Alzheimer’s-like dementia Wistar rats (male, 300–350 g, n = 10/10)	50/100 mg/kg, orally, 21 days	①
Durairajan et al. (2012)	TgCRND8 mice (Two-month-old, n = 6/6)	25/100 mg/kg, orally, 6 months	④
He et al. (2017)	APP/PS1 transgenic mice (120-day, male, n = 10/10)	50/100 mg/kg, Intragastric administration, 14 days	①②③
Huang et al. (2017)	3 × Tg-AD mice (4 months, male,6; female,6, n = 12/12)	50/100 mg/kg/day, orally, 4 months	①②③④
Hussien et al. (2018)	the heavy metals induced AD Sprague-Dawley rats weighing (adult, 170–220 g, female, n = 10/10)	50 mg/kg/day, orally, 30 days	①③④
Kumar et al. (2016)	ICV-STZ induced Alzheimer’s-like dementia Wistar rats (200–250 g, male, n = 7/7)	25/50/100 mg/kg, orally, 21 days	①②③
Liang et al. (2021)	3 × Tg AD mice. (four-month-old, male, n = 12/12)	50/100 mg/kg, orally, 4 months	①②③④
Lin et al. (2020)	B6C3-Tg (APPswePSEN1dE9)/Nju double transgenic mice (4.5 months, male,6; female,6)	100 mg/kg/day, intragastrically, 3 months	①②③④
Mangrulkar et al. (2013)	Colchicine induced cognitive impairment Swiss Albino Mice (20–25 g, n = 6/6)	5/10/20/40 mg/kg, orally, 21 days	①②
Patil et al. (2015)	Ethanol treated Wistar rats (adult, 200–250 g, male, n = 6/6)	25/50/100 mg/kg, orally administered, 45 days	①②
Wang et al. (2021b)	APP/PS1 transgenic mice (16 weeks, n = 10/10)	100 mg/kg/day, Intragastric injection, 3 weeks	①②③
Wu et al. (2021)	APP/PS1 transgenic mice (Six-month-old, male, n = 15/15)	260 mg/kg, orally, 3 months	①②④
Yang and Wang (2022)	APP/PS1 mice (4 months, male, n = 10/10)	50/100 mg/kg. Intragastric administration, 4months	①②③
Ye et al. (2021)	3 × Tg AD mice (4-month male, n = 12/12	100 mg/kg, orally, 4 months	①②③
Zhu and Qian (2006)	Sprague-Dawley rat model of AD established by injecting Aβ(1–40) (adult, male, 220-220 g, n = 6/6)	50 mg/kg, intragastrically, 14 days	①③

①: escape latency; ②: the duration in platform quadrant; ③: platform crossing number; ④: APP.

### 3.2 Study quality evaluation and risk of bias assessment

Study Quality Assessment Table 2 shows the methodological quality assessment of the 19 included studies, with study quality scores ranging from 3 - 6 out of 10. No studies were considered to be at low risk of sequence generation, allocation concealment (selection bias), blinded intervention and randomized outcome assessment (detection bias).15 studies were considered to be at low risk of baseline characteristics (selection bias). 15 studies were judged to be at low risk of randomized housing (performance bias). 15 studies were judged to have a low risk of incomplete outcome data (attrition bias). However, only 6 studies were considered to have a low risk of blinding assessment of outcome (detection bias). All 19 studies were considered to be at low risk of selective outcome reporting and other sources of bias.

**TABLE 2:** The methodological quality assessments of 19 included studies.

Study	1	2	3	4	5	6	7	8	9	10	Total
Bhutada et al. (2011)	NC	Y	NC	NC	NC	NC	NC	Y	Y	Y	4
Cai et al. (2018)	NC	Y	NC	Y	NC	NC	NC	Y	Y	Y	5
Chen et al. (2017)	NC	Y	NC	Y	NC	NC	NC	Y	Y	Y	5
Chen et al. (2020b)	NC	Y	NC	Y	NC	NC	NC	Y	Y	Y	5
de Oliveira et al. (2016)	NC	Y	NC	Y	NC	NC	NC	NC	Y	Y	4
Durairajan et al. (2012)	NC	Y	NC	Y	NC	NC	Y	Y	Y	Y	6
He et al. (2017)	NC	Y	NC	Y	NC	NC	NC	Y	Y	Y	5
Huang et al. (2017)	NC	Y	NC	NC	NC	NC	Y	Y	Y	Y	5
Hussien et al. (2018)	NC	Y	NC	Y	NC	NC	NC	Y	Y	Y	5
Kumar et al. (2016)	NC	Y	NC	Y	NC	NC	NC	Y	Y	Y	5
Liang et al. (2021)	NC	Y	NC	Y	NC	NC	Y	Y	Y	Y	6
Lin et al. (2020)	NC	Y	NC	Y	NC	NC	Y	Y	Y	Y	6
Mangrulkar et al. (2013)	NC	Y	NC	Y	NC	NC	NC	NC	Y	Y	4
Patil et al. (2015)	NC	Y	NC	Y	NC	NC	NC	Y	Y	Y	5
Wang et al. (2021b)	NC	NC	NC	NC	NC	NC	NC	Y	Y	Y	3
Wu et al. (2021)	NC	Y	NC	Y	NC	NC	NC	Y	Y	Y	5
Yang and Wang (2022)	NC	NC	NC	Y	NC	NC	NC	NC	Y	Y	3
Ye et al. (2021)	NC	NC	NC	Y	NC	NC	Y	Y	Y	Y	5
Zhu and Qian (2006)	NC	NC	NC	NC	NC	NC	Y	NC	Y	Y	3

1—sequence generation; 2—baseline characteristics; 3—allocation concealment; 4—random housing; 5—blinded intervention; 6—random outcome assessment; 7—blinded assessment of outcome; 8—incomplete outcome data; 9—selective outcome reporting; 10—other sources of bias. Y, yes; N, no; NC, unclear.

### 3.3 Results of the meta-analysis

#### 3.3.1 Effect of berberine on cognitive function in AD models: Escape latency of MWM

**FIGURE 2:**
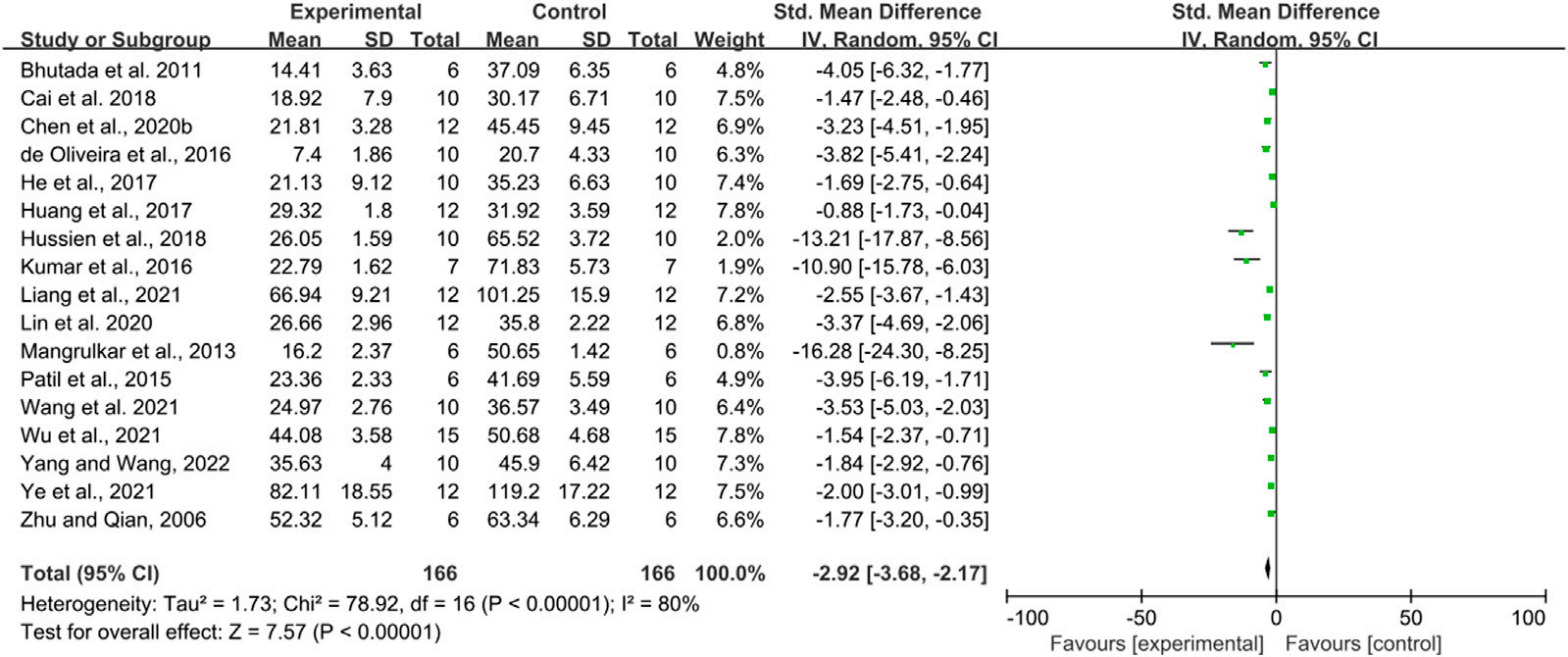
The effect of berberine on the escape latency of MWM.

Seventeen studies with a total of 332 animals used escape latency as an outcome indicator and all reported a positive effect of berberine in reducing escape latency. However, there was a high heterogeneity between studies (*p* < 0.00001, I^2^ = 80%), so a random effects model was performed for analysis. According to the results, in terms of reducing escape latency, the intervention group had a significant effect in reducing escape latency compared to the control group (SMD = −2.92, 95% CI: 3.68, −2.17, *p* < 0.00001) (Figure 2).

Due to heterogeneity, we further performed subgroup analyses based on animal species (mice, rats), intervention dose (<100 mg/kg, ≥100 mg/kg), duration of intervention (<14, 14–30, >30 days), and route of administration (oral administration and gavage administration). The results are shown below Table 3.

**TABLE 3:** Subgroup analyses of escape latency.

Subgroup	SMD	LL	HL	Df	I^2^	Z	P
Animal species							
Rats	−5.40	−7.79	−3.01	5	84	4.44	*p* < 0.00001
Mice	−2.22	−2.87	−1.57	10	71	6.70	*p* = 0.0002
Dosage							
<100 mg/kg	−9.95	−19.83	−0.07	2	94	1.97	*p* < 0.00001
≥100 mg/kg	−2.55	−3.18	−1.92	13	71	7.89	*p* < 0.0001
Time							
<14 days	−1.62	−2.27	−0.97	2	0	4.88	*p* < 0.00001
14–30 days	−7.09	−9.86	−4.31	5	84	5.01	*p* < 0.00001
>30 days	−2.23	−2.89	−2.17	7	64	6.58	*p* < 0.00001
Route							
Oral administration	−2.53	−3.42	−1.64	7	71	5.57	*p* < 0.001
Intragastric administration	−3.48	−4.76	−2.21	16	85	7.57	*p* < 0.00001

Table 3 demonstrated that different animal species, dose, duration of intervention and route of administration significantly shortened the escape latency (*p* < 0.05) compared to controls.

The subgroup analysis observed that mice had slightly reduced heterogeneity (I^2^ = 71%), while rats did not (I^2^ = 84%). For dose, medication doses ≥100 mg/kg had mildly reduced heterogeneity (I^2^ = 71%), whereas <100 mg/kg did not (I^2^ = 94%). Heterogeneity was significantly reduced for berberine interventions <14 days (I^2^ = 0), slightly reduced for >30 heterogeneity (I^2^ = 64%), no reduction in heterogeneity for 14–30 days (I^2^ = 84%). For route of administration, a slight decrease in heterogeneity with oral administration of berberine (I^2^ = 71%) and no decrease with gavage administration (I^2^ = 84%).

#### 3.3.2 Effects of berberine on cognitive function in AD models: Time spent in the MWM platform quadrant

**FIGURE 3:**
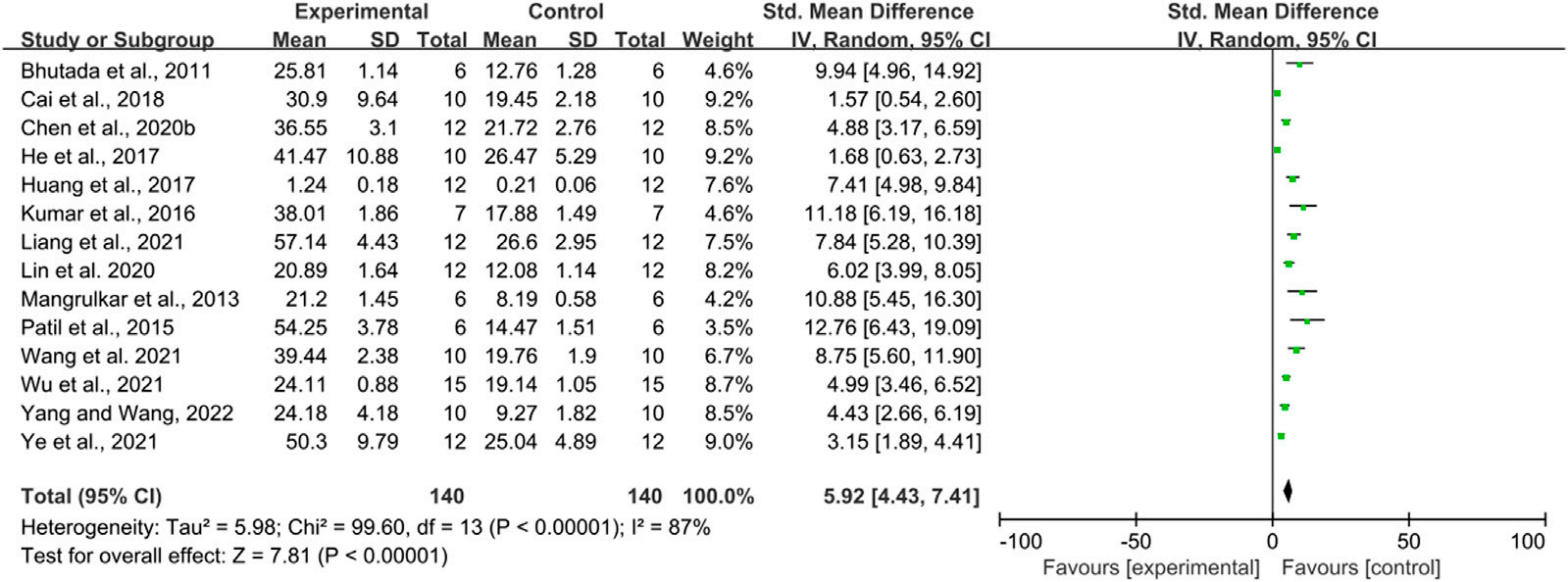
The effect of berberine on Time Spent in the Platform Quadrant of MWM.

Fourteen studies used time spent in the platform quadrant as an outcome measure, totaling 280 mice. There was a high degree of heterogeneity between studies (I^2^ = 87%, *p* < 0.00001), so a random-effects model was performed for analysis. Meta-analysis showed a significant difference between the intervention and control groups in terms of time spent in the platform quadrant. (SMD = 5.92, 95% CI (4.43, 7.41), *p* < 0.00001) (Figure 3).

Due to heterogeneity, we further performed subgroup analyses based on animal type (mice and rats), duration of intervention (<14, 14–30, >30 days), and route of administration (oral administration and gavage administration). The results are shown below Table 4.

**TABLE 4:** Subgroup analyses of time spent in the platform quadrant.

Subgroup	SMD	LL	HL	Df	I^2^	Z	P
Animal species							
Rats	11.08	8.00	14.16	2	0	7.05	*p* < 0.00001
Mice	5.09	3.64	6.54	10	87%	6.88	*p* < 0.00001
Time							
<14 days	1.62	0.89	2.36	1	0%	4.32	*p* < 0.0001
14–30 days	9.76	7.61	11.92	3	0%	8.87	*p* < 0.00001
>30 days	5.6	4.29	6.91	7	71%	8.39	*p* < 0.00001
Route							
Oral administration	7.26	5.01	9.52	6	81%	6.31	*p* < 0.00001
Intragastric administration	4.77	2.87	6.67	6	88%	4.92	*p* < 0.00001

Table 4 demonstrated that different animal species, duration of intervention and route of administration significantly increased time spent in the platform quadrant (*p* < 0.05) compared to controls.

The subgroup analysis observed that a significant decrease in heterogeneity in rats (I^2^ = 0%), and no decrease in heterogeneity in mice (I^2^ = 87%). For duration of intervention, a significant reduction in heterogeneity for berberine interventions <14 days and interventions 14–30 days (I^2^ = 0) and a slight reduction in heterogeneity >30 (I^2^ = 71%). For route of administration, subgroup analyses found no decrease in heterogeneity for both oral (I^2^ = 81%) and gavage administration of berberine (I^2^ = 87%).

#### 3.3.3 Effects of berberine on cognitive function in AD models: Number of MWM platform crossings

**FIGURE 4:**
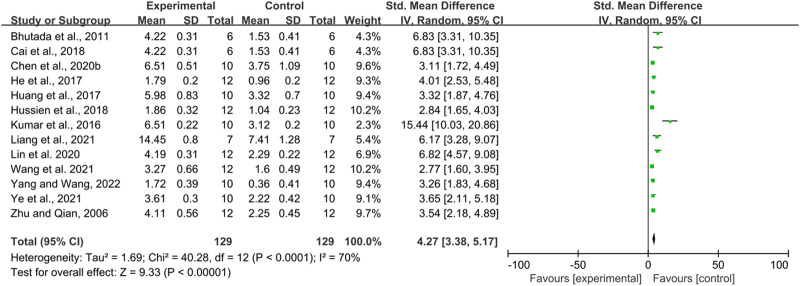
The effect of berberine on Platform crossover numbers of MWM.

Thirteen studies used the number of platform crossings as an outcome measure, totaling 258 mice. There was a high degree of heterogeneity between the studies (I^2^ = 70%, *p* < 0.00001), so a random effects model was performed for analysis. Meta-analysis showed a significant difference between the intervention and control groups in terms of the number of platform crossings compared to the control group (SMD = 4.27, 95% CI (3.38, 5.17); *p* < 0.00001). (Figure 4).

Due to the heterogeneity, we further performed subgroup analyses based on animal type (mice and rats), intervention dose (<100 mg/kg, ≥100 mg/kg), duration of intervention (<14, 14–30, >30 days), and route of administration (oral administration and gavage administration). The results are shown below Table 5.

**TABLE 5:** Subgroup analyses of platform crossover numbers.

Subgroup	SMD	LL	HL	Df	I^2^	Z	P
Animal species							
Mice	3.94	3.12	4.77	8	53	9.39	*p* < 0.00001
Rats	5.90	2.97	8.83	3	87	3.95	*p* < 0.0001
Dosage							
<100 mg/kg	4.02	2.38	5.66	6	84	4.81	*p* < 0.00001
≥100 mg/kg	4.21	0.92	7.5	1	78	2.51	*p* = 0.01
Time							
<14 days	2.42	0.87	3.97	2	75	3.05	*p* = 0.002
14–30 days	7.28	3.33	11.24	3	86	3.61	*p* = 0.0003
>30 days	3.68	2.79	4.57	5	56	8.12	*p* < 0.00001
Route							
Oral administration	5.19	3.26	7.12	5	81	5.27	*p* < 0.0001
Intragastric administration	3.87	2.95	4.79	6	57	8.21	*p* < 0.00001

Table 5 demonstrated that different animal species, dose, duration of intervention and route of administration significantly increased the number of platform crossings compared to the control group (*p* < 0.05).

The subgroup analysis observed that a slight decrease in heterogeneity in mice (I^2^ = 53%). No change in heterogeneity in rats (I^2^ = 87%). For dose, no heterogeneity reduction with doses <100 mg/kg (I^2^ = 84%) or ≥100 mg/kg (I^2^ = 78%). For duration of intervention, no reduction in heterogeneity for berberine interventions <14 days (I^2^ = 75%) and 14–30 days (I^2^ = 80%), with a slight reduction in heterogeneity for berberine interventions >30 days (I^2^ = 56%). For route of administration, a slight decrease in heterogeneity with berberine gavage administration (I^2^ = 57%) and no decrease with gavage administration (I^2^ = 81%).

#### 3.3.4 Effect of berberine on β-amyloid precursor protein in AD models

**FIGURE 5:**
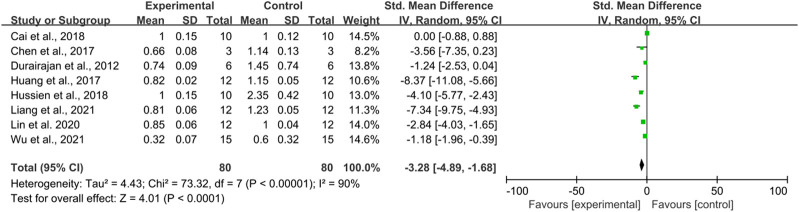
The effect of berberine on APP.

There were eight studies using β-amyloid precursor protein as an outcome Indicator, totaling 160 mice. There was a high heterogeneity among the studies (I^2^ = 90%, *p* < 0.0001), and therefore a random-effects model was performed for the analysis. Meta-analysis showed that there was a significant difference between the intervention and control groups compared to the control group in terms of APP (SMD = −3.28, 95% CI (−4.89, −1.68); *p* < 0.0001). (Figure 5).

Due to heterogeneity, we further performed subgroup analyses based on animal type (mice and rats), duration of intervention (≤30, >30 days), and route of administration (oral administration and gavage administration). The results are shown below Table 6.

**TABLE 6:** Subgroup analyses of APP.

Subgroup	SMD	LL	HL	Df	I^2^	Z	P
Animal species							
Rats	−4.01	−5.54	−2.49	1	0	5.15	*p* < 0.00001
Mice	−3.12	−4.95	−1.30	5	92%	3.35	*p* = 0.008
Time							
≤30 days	−1.96	−3.95	0.03	2	85%	1.93	*p* = 0.05
>30 days	−4.61	−7.53	−1.82	3	93%	3.21	*p* = 0.001
Route							
Oral administration	−1.59	−3.19	0.01	3	81%	1.94	*p* = 0.05
Intragastric administration	−3.28	−4.89	−1.69	3	94%	2.93	*p* = 0.003

Table 6 demonstrated that different animal species significantly reduced APP levels compared to the controls (*p* < 0.05), whereas there was no difference for berberine at ≤30 days (*p* = 0.05) of dosing and oral administration (*p* = 0.05).

The subgroup analysis observed that heterogeneity was significantly reduced in rats (I^2^ = 0%) but unchanged in mice (I^2^ = 92%). For duration of intervention, no heterogeneous reduction in berberine intervention ≤30 days (I^2^ = 85%) or berberine intervention >30 days (I^2^ = 95%). For route of administration, there was no decrease in heterogeneity for berberine administered by gavage (I^2^ = 94%) or by oral administration (I^2^ = 81%).

### 3.4 Publication bias

**FIGURE 6:**
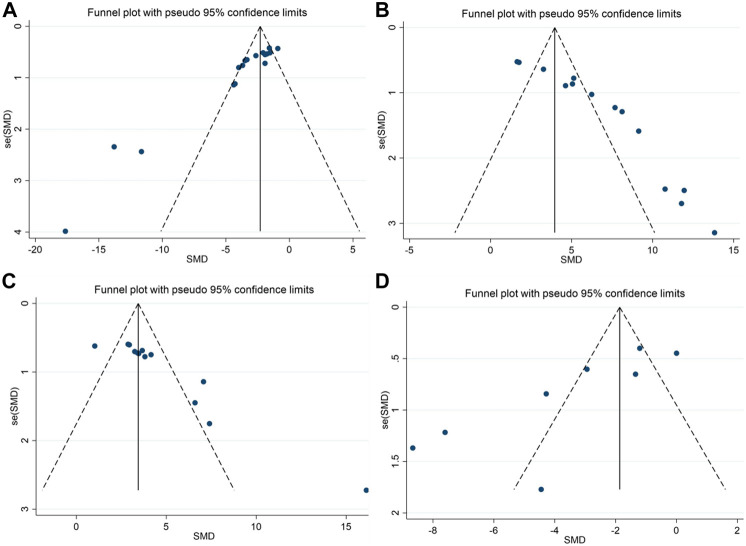
Funnel plots showing the effect of berberine on cognitive function and APP in AD models; **(A)** escape latency; **(B)** the duration in platform quadrant; **(C)** platform crossing number; **(D)** APP.

We performed publication bias tests for the four outcomes using funnel plots (Figure 6) and Egger’s test. The results show that scatters such as APP, escape latency, platform quadrant duration, and number of cross-platforms are largely outside the scope of the funnel plot, demonstrating asymmetry. Egger’s test showed potential evidence of publication bias in escape latency (*p* = 0.000), duration of plateau quadrant (*p* = 0.000), number of plateau crossings (*p* = 0.000), and APP (*p* = 0.009), suggesting the presence of publication bias. To assess the effect of publication bias for the outcomes (APP, escape latency, platform quadrant duration, and number of platform crossings, APP), we used a trim-and-fill approach. The results showed that the robustness of these outcomes was not significantly affected by publication bias (Table 7).

**TABLE 7:** Results from Egger’s test and trim and fill analysis.

Outcomes	Egger’s test	Before trim and fill			After trim and fill		
	*p*-value	*p*-value	Est (F/R)	No. Studies	*p*-value	Est (F/R)	No. Studies
Escape latency	0.000	0.000	−2.301/-3.160	17	0.000	0.100/0.042	17
The duration in platform quadrant	0.000	0.000	3.960/6.322	14	0.000	29.287/47.018	20
Platform crossing number	0.000	0.000	3.438/4.171	13	0.000	19.227/20.115	18
APP	0.009	0.000	−1.867/-3.509	8	0.000	0.155/0.030	8

Est, total effect sizes; F/R, fixed effect model/random-effects model; No., number.

### 3.5 Meta-regression analysis

Publication bias was measured by escape latency as an outcome measure, and we performed univariate meta-regression to explore the sources of heterogeneity, and multivariate meta-regression with year of publication, study sample size, sex of animals, animal species (mice and rats), method of administration (oral and gavage), and duration of administration as covariates. Regression to look for sources of heterogeneity.

In the meta-regression analysis, heterogeneity in escape latency could not be determined using year of publication (*β* = 0.14, I^2^ = 83.55%, *r* ^2^ = −18.16%, *p* = 0.531), study sample size (*β* = 0.52, I^2^ = 81.87%, *r* ^2^ = 10.28%, *p* = 0.135), animal species (*β* = −0.73, I^2^ = 82.60%, and *r* ^2^ = −25.34%, *p* = 0.329), animal sex (*β* = −0.94, I^2^ = 83.58%, *r* ^2^ = −20.34%, *p* = 0.417), method of administration (*β* = −1.05, I^2^ = 83.52%, *r* ^2^ = −11.67%, *p* = 0.583) and duration of administration (*β* = 0.02, I^2^ = 82.83%, r^2^ = 1.33%, *p* = 0.182) were explained. These covariates were not associated with heterogeneity (Supplementary Figures S1–S6).

### 3.6 Sensitivity analysis

**FIGURE 7:**
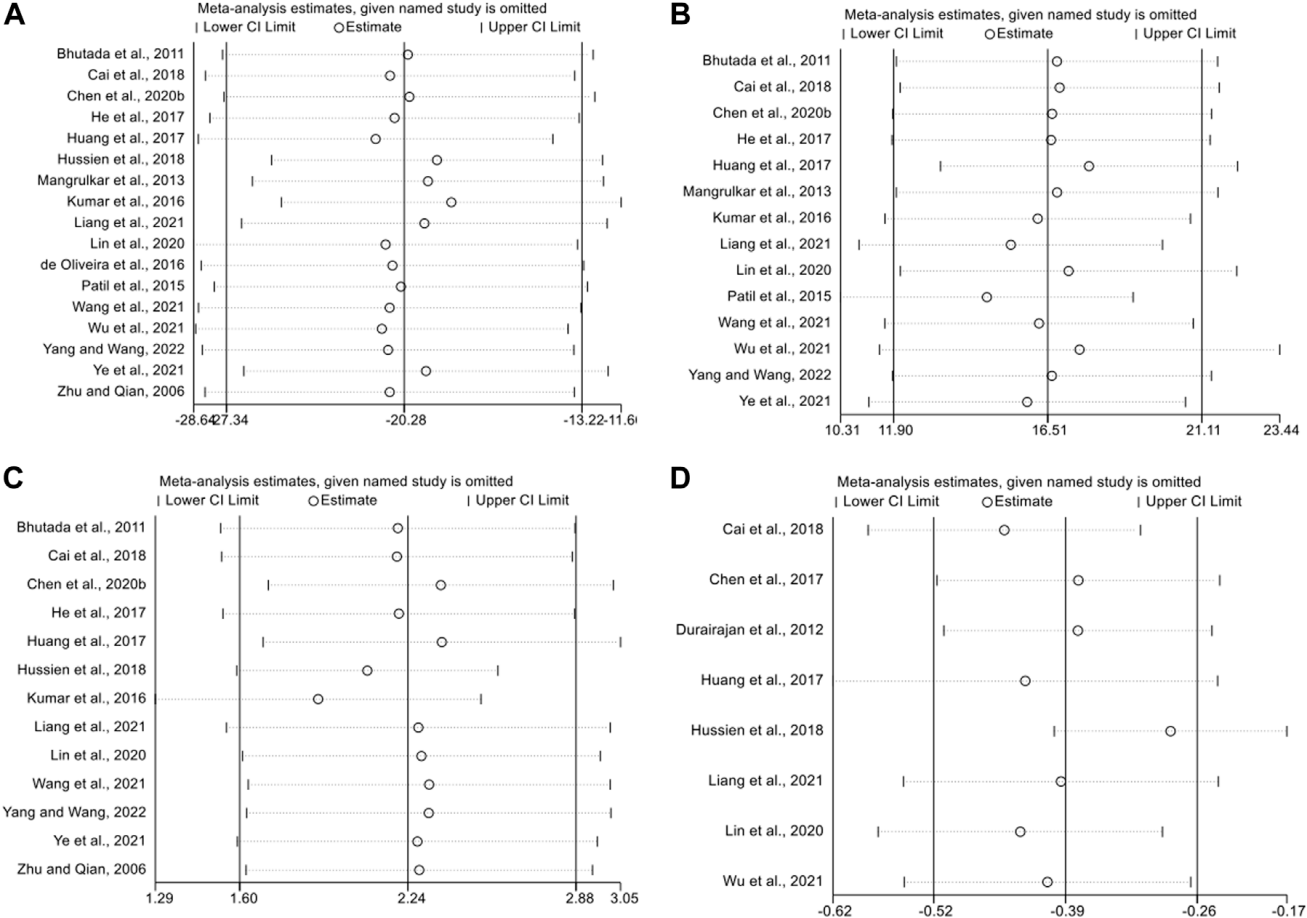
The sensitivity analysis of included studies; **(A)** escape latency; **(B)** the duration in platform quadrant; **(C)** platform crossing number; **(D)** APP.

We performed sensitivity analyses to test the stability of the meta and to identify sources of heterogeneity. We performed sensitivity analyses based on escape latency, number of platform crossings, time in the target quadrant, and APP outcome expression. After consecutively removing each study, the effects of the remaining studies were within the 95% CI of the total effect. This indicates that the sensitivity level of the meta-analysis was low and the results were stable and reliable (Figure 7).

## 4 Discussion

To our knowledge, this is the inaugural systematic evaluation and meta-analysis investigating the impact of berberine on cognitive function and APP in an AD animal model, employing the MWM water maze test and APP as outcome indicators. Our findings suggest that berberine has a potential impact on improving cognitive function and APP expression in AD animal models. Berberine holds promise in enhancing cognitive function and reducing APP expression in AD animal models.

Yuan et al. published the first review on the effects of berberine on AD in animal studies in 2019 (Yuan et al., 2019). The review highlighted consistent positive effects of berberine on memory deficits across various animal models, indicating its therapeutic potential for AD. However, Yuan’s review had limitations, including the inclusion of studies without experimental and animal behavioral assessments. While the overall effects were evident, the microscopic mechanisms remain unclear, necessitating further research to elucidate biochemical details and specific drug targets. Additionally, the review lacked a meta-analysis, a gap addressed by our study.

Our studies exhibited a significant degree of heterogeneity. Animal studies are typically designed to be more exploratory and heterogeneous than clinical trials. Subgroup analyses were conducted to identify the sources of heterogeneity. The subgroup analyses considered animal species, intervention duration, administration route, and dose. Following subgroup analysis, berberine’s positive effects on AD animals persisted, and some heterogeneity was effectively explained. For instance, compared to controls, berberine intervention for <14 days (SMD = 1.62, 95% CI (0.89, 2.36), *p* < 0.0001), I^2^ = 0%) and 14–28 days (SMD = 9.76, 95% CI (7.61, 11.92), *p* < 0.00001), I^2^ = 0%) significantly extended the duration in the platform quadrant, indicating that intervention time might contribute to heterogeneity. However, despite further sensitivity analyses and meta-regression analyses, the heterogeneity between studies based on escape latency and APP could not be fully elucidated, and more studies are still needed to provide more precise evidence. Eight AD models were included in this study: four transgenic animal models and four drug-induced animal models. APP/PS1 double transgenic mice were generated from two vectors. One vector encodes the APP gene carrying the Swedish (KM 670/671 NL) mutation, and the other encodes the FAD-linked PSEN 1 gene without exon 9 (dE 9) (Yokoyama et al., 2022). These mice exhibit Aβ deposition starting at 6 months, escalating at 9 months, and display impairment by 12 months, marked by an increase in neurogenic and reactive astrocytes (Kamphuis et al., 2012) with plaque deposition. APP/PS1/tau 3 × Tg-AD mice might surpass APP/PS1 double transgenic mice due to the latter’s comparatively limited pathology in hyperphosphorylated tau proteins, a significant feature associated with AD dementia severity (Yang et al., 2018). These two types of mice constituted the most utilized AD models in this study. Activated microglia in TgCRND 8 mice exhibit early accumulation of amyloid plaques at 3 months, followed by a strong astrocyte response shortly thereafter (Yokoyama et al., 2022). The B6C3-Tg transgenic mouse is an "amyloid-only" strain generating viable brain amyloid deposits and cognitive deficits (Finnie et al., 2017). While injections of Aβ, streptozotocin, colchicine, or other drugs are relatively simple and stable, the evidence of neurodegeneration in these models varies, presenting mixed research outcomes. There exists a disparity between drug-induced AD models and the intricate, multimodal pathology of human AD. Strategies divergent from the current approach need consideration to bridge this gap (Nazem et al., 2015). In essence, the existence of diverse AD models may engender substantial result heterogeneity. Experimental biases in water maze tests (Othman et al., 2022) and differences in study protocol designs, among other dissimilarities, could also contribute to this heterogeneity.

A substantial portion of the research on AD treatment with Traditional Chinese Medicine (TCM) has focused on herbs like Panax ginseng (Razgonova et al., 2019), curcumin (Bhat et al., 2019), active constituents of herbs such as polysaccharides (Zhang et al., 2022), epimedium glycoside Icariin (Yu et al., 2022), evodiamine (Pang et al., 2022) and specific therapies such as Tai Chi (Park et al., 2023), acupuncture (Cai et al., 2019), aiming to ameliorate memory and cognitive deficits in AD models. These approaches operate through diverse mechanisms, including regulation of β-amyloid and tau proteins, amelioration of cognitive deficits by preventing synaptic degeneration and neuroinflammation in AD, and modulation of AD pathology via mechanisms like antioxidant activity, anti-neuroapoptosis, or autophagy. Other nonpharmacologic interventions play an important role in improving AD development. Studies have shown that exercise increases AMPK activity and upregulation of the PGC-1α/FNDC5/BDNF pathway, which are involved in mediating the beneficial effects of exercise on Aβ-induced learning and memory deficits in mice (Azimi et al., 2018). It also prevents the decline in hippocampus-dependent cognitive function and Aβ deposition in early AD progression by modulating microglia-mediated neuroinflammation and oxidative stress (Zhang et al., 2019). Exercise programs combining aerobic (swimming) and resistance exercise, or using moderate intensity running have better results. This places greater demands on the intensity, duration, and construction of study protocols, and the exact mechanisms by which exercise training improves cognitive activity are not fully understood. There is also an acupuncture therapy that has a higher overall benefit in combination with conventional medications or other therapies (Jia et al., 2017; Wang et al., 2021a; WuLi et al., 2021). However, methodological problems from clinical trials and animal experiments affect the strength of the evidence. Therefore, more randomized clinical trials and better study designs are needed. Compared with other pharmacologic interventions, berberine has a variety of potential multi-targeted therapeutic effects for the treatment of AD, including anti-neuroinflammation, inhibition of endoplasmic reticulum stress, and improvement of the cholinergic system (Tian et al., 2023). The unique advantage of safranin is that it has been used as an anti-inflammatory and bacteriostatic drug for many years in the clinic, with effectiveness and safety (Song et al., 2020). It has been found that safranin can cross the blood-brain barrier (Wu et al., 2022b), and play a therapeutic role in central nervous system diseases. This provides a theoretical basis for the study of flavosol intervention in the treatment of AD.

AD pathology, observed microscopically in brain tissue, presents two abnormal structures: extracellular amyloid plaques and intraneuronal neurofibrillary tangles. Aβ precedes tau in the pathogenesis of AD, initiating tau’s conversion from a normal to a toxic state. Thus, early detection of plaques, tangles, and cognitive deficits, coupled with intervention to disrupt the biochemical pathways they trigger, could facilitate successful AD therapy (Bloom, 2014). The transmembrane glycoprotein, Amyloid Precursor Protein (APP), widely present in cell membranes across various tissues, significantly contributes to the Aβ pathogenic pathway. Tau strongly promotes BACE1 expression and Aβ production when interacting with APP (Zhang et al., 2021b). Aβ is derived from amyloid by sequential cleavage of β and γ-secretases (Crews et al., 2010). In the non-amyloidogenic pathway, α-secretase cleaves APP, generating N-terminal secreted APP (sAPPα) and an 83-amino-acid C-terminal fragment (CTF) (C83), which, upon further cleavage by γ-secretase, produces the 3 kDa product (P3) and the APP intracellular domain (AICD) (Wang et al., 2017). A small amount of APP is cleaved by β-secretase at Asp 1 (β-site) and Glu 11 (Crews et al., 2010). Glu 11 is the major β-cleavage site that produces the 89-amino acid CTF (C89), which is further cleaved by γ-secretase to produce the truncated Aβ1-40/42. While β-site APP cleavage enzyme 2 (BACE2) shares homology with BACE1, its role differs; it acts as a θ-secretase, cleaving APP within the Aβ structural domain, impeding Aβ production (Liu et al., 2013). Our study showed that berberine reduced APP expression in an animal model of AD, suggesting that berberine can process APP to reduce Aβ. The mechanism may be to affect the hyperphosphorylation of BACE1, Tau protein, or the terminal fragment of APP (C99) to regulate Aβ. Berberine is involved in regulating APP modification, which may inhibit Aβ production through BACE1 inhibition and regulation of γ-secretase substrates. This can adjust the preference of cleavage sites that favor Aβ reduction. However, the precise downstream mechanisms of berberine-induced APP downregulation necessitate further exploration. Additionally, in the Morris water maze, berberine treatment exhibited favorable effects on cognitive function in an AD animal model, manifesting in reduced escape latencies, prolonged platform quadrant durations, and increased platform crossings. Aβ levels in the hippocampus seemed to correlate with spatial cognitive deficits in AD (Wu et al., 2022a). The observed enhancement in spatial cognitive function due to berberine may primarily result from attenuated hippocampal Aβ. Protein phosphatase 2A (PP-2A) modulates Aβ levels by regulating APP phosphorylation and β- and γ-secretase activities (Zhang et al., 2015). It suggesting that berberine may be a good multi-targeted drug that can modulate AD related substances tau, PP-2A, Aβ, APP, or BACE-2. However, identifying the specific target among multiple targets that plays a pivotal role in berberine modulation remains a focal point for future research.

This study exhibits several limitations. First, the included studies used a variety of AD models, and the total number of studies and total sample size were relatively small, which may have affected the effectiveness of the berberine intervention. Second, in terms of study quality, the method of randomized allocation was unclear and none of the studies clearly described the methods used for generation of allocation sequences, hiding of allocation sequences, blinded interventions, and assessment of randomized outcomes. Subsequent studies should aim for more refined and comprehensive approaches in terms of high-quality research. Third, although we attempted to use pre-designed subgroup analyses, meta-regression to explore sources of heterogeneity, it seems to be ineffective. Various experimental details of different study protocols and intervention processes in animal experiments may also be the key potential factors leading to a high degree of heterogeneity. More attention should be paid to the rigor of study design and the provision of sufficient experimental information in future studies. Fourth, we did not conduct further meta-analysis on the relevant indicators because the available data for some indicators only existed in individual studies, and more attention needs to be paid to these indicators in the future.

## 5 Conclusion

Berberine improved cognitive dysfunction and decline of β-amyloid precursor protein in AD animals. Berberine showed significant memory-improving activity in several animal models of memory deficits by mechanisms including downregulation of APP-associated protein expression, modulation of Aβ, and effects on BACE1 and tau phosphorylation, but the validity of the findings may be affected by heterogeneity and publication bias. More rigorous experimental designs and more comprehensive studies are needed to test the protective effects of berberine in animal models of AD in the future.

## Data Availability

The original contributions presented in the study are included in the article/Supplementary Materials, further inquiries can be directed to the corresponding author.
