# Supplementary material for: Effect of berberine on cognitive function and β-amyloid precursor protein in Alzheimer’s disease models: a systematic review and meta-analysis
Source: Front Pharmacol. 2024 Jan 16;14:1301102. doi: 10.3389/fphar.2023.1301102 (PMC10824956; doi:10.3389/fphar.2023.1301102)
Supplement: Supplementary file 1 [file Table1.DOCX]

Supplementary Material

Search strategies

601 of PubMed

| ID | Search | Results |
| --- | --- | --- |
| #1 | (Alzheimer Disease [MeSH Terms]) OR (Dementia [MeSH Terms]) OR (Cognition [MeSH Terms]) OR (Alzheimer) OR (AD) OR (cognitive) | 2528406 |
| #2 | ("Berberine"[MeSH Terms] OR "Umbellatine"[Title/Abstract] | 4077 |
| #3 | #1 AND #2 | 601 |

753 of Embase

| NO. | Search | Results |
| --- | --- | --- |
| #1 | 'alzheimer disease'/exp OR 'dementia'/exp OR 'cognition'/exp OR 'alzheimer' OR 'ad' OR 'cognitive' | 3821374 |
| #2 | 'berberine'/exp OR 'berberine' OR 'umbellatine'/exp OR 'umbellatine' | 11531 |
| #3 | #1 AND #2 | 753 |

846 of Medline

| ID | Search | Results |
| --- | --- | --- |
| #1 | (Alzheimer disease or dementia or cognition or Alzheimer or AD or cognitive).af. | 2428389 |
| #2 | (berberine or Umbellatine).af. | 7665 |
| #3 | #1 AND #2 | 846 |

25 of Cochrane Library

| ID | Search | Hits |
| --- | --- | --- |
| #1 | (Alzheimer disease OR dementia OR cognition OR Alzheimer OR AD OR cognitive):ti,ab,kw | 172097 |
| #2 | (berberine):ti,ab,kw OR ("umbellatine"):ti,ab,kw | 394 |
| #3 | #1 AND #2 | 25 |

313 of Web of science

| ID | Search | Results |
| --- | --- | --- |
| #1 | ALL=(Alzheimer disease OR dementia OR cognition OR Alzheimer OR AD OR cognitive) | 887992 |
| #2 | ALL=(Berberine OR Umbellatine) | 6668 |
| #4 | #1 AND #2 | 313 |

Supplemental Fig.1 Univariate meta-regression based on publication year as covariate


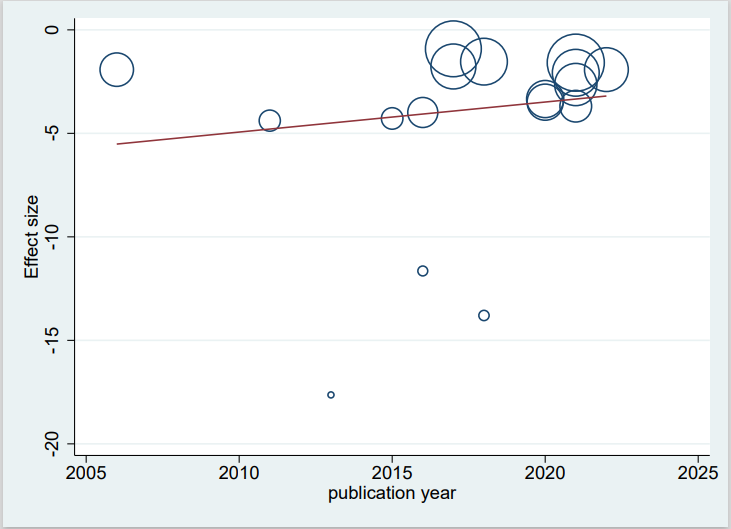


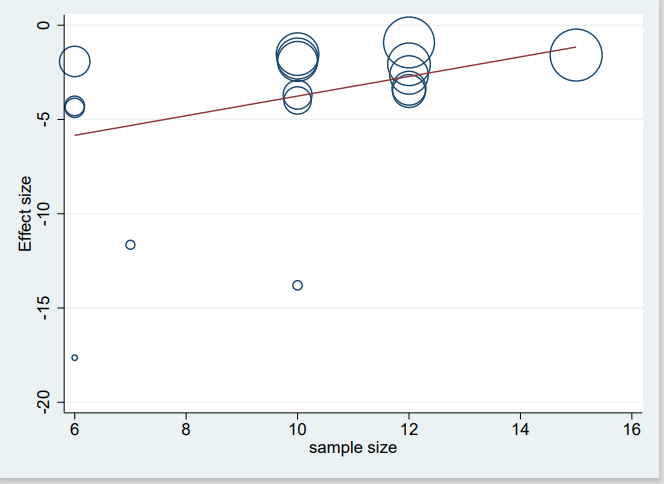
Supplemental Fig.2 Univariate meta-regression based on study sample size as covariate


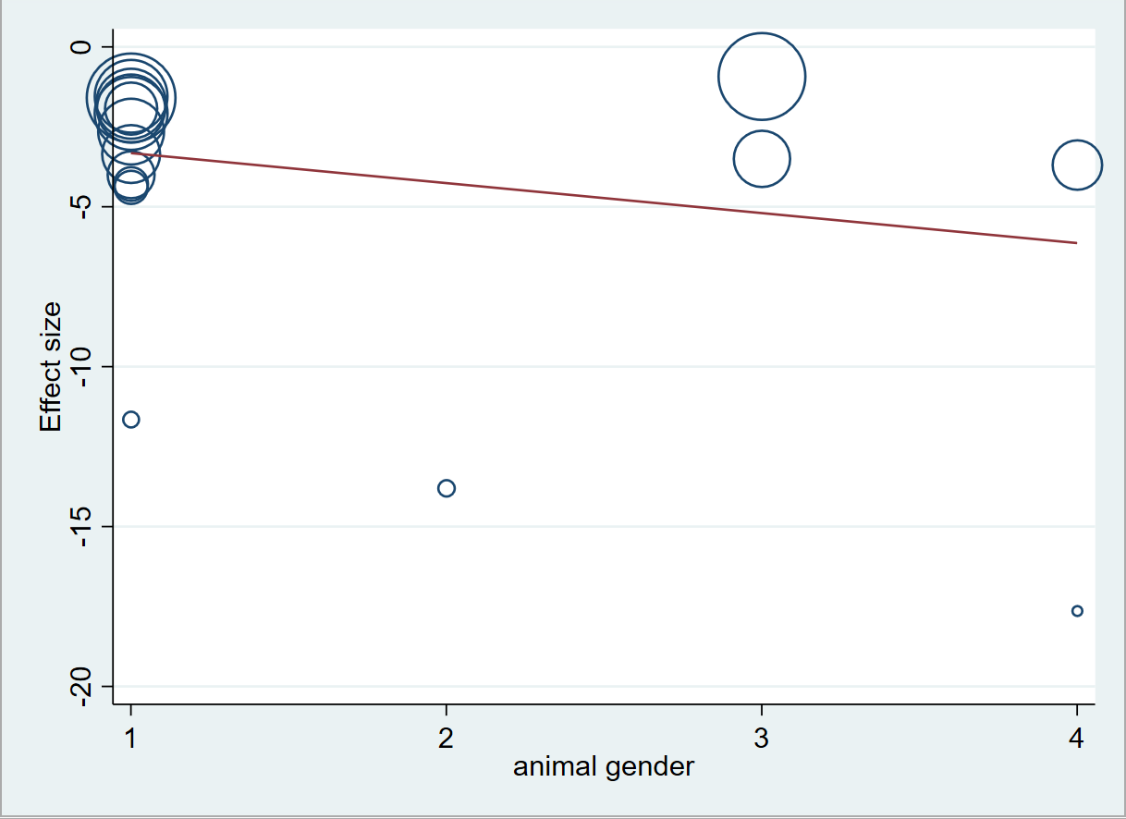
Supplemental Fig.3 Univariate meta-regression based on animal gender as covariate


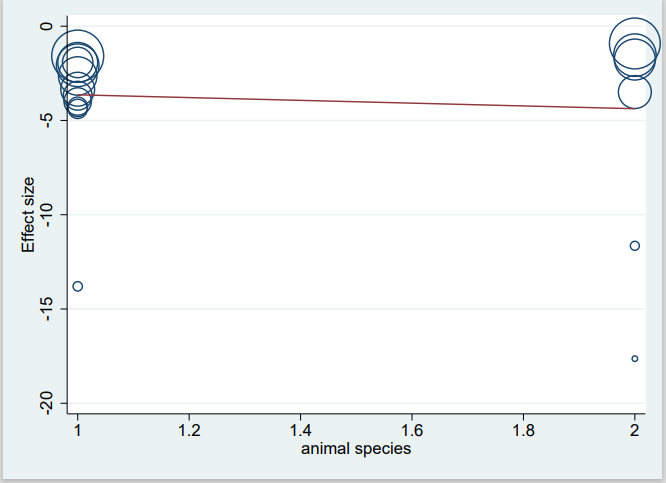
Supplemental Fig.4 Univariate meta-regression based on animal species size as covariate

Supplemental Fig.5 Univariate meta-regression based on route of administration size as covariate


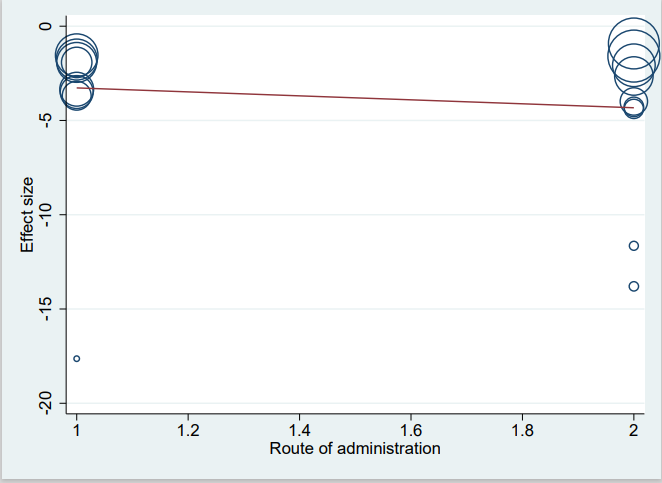


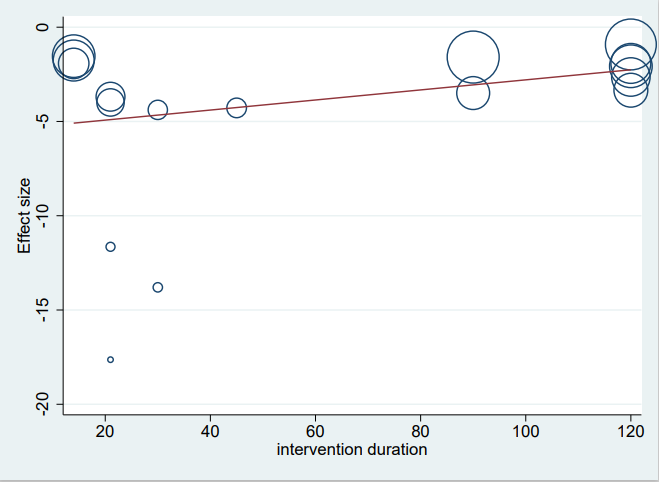
Supplemental Fig.6 Univariate meta-regression based on intervention duration size as covariate
